# Reading the Scriptures

**Published:** 1890-06-14

**Authors:** 


### Words of Consolation.

### READING THE SCRIPTURES.

There are many kinds of reading very undesirable for sick persons, such as novels, plays, and foolish jest-books, which only excite the mind and lead it astray from God, who has sent the illness to wean them from the world and fit them for His service.

The Holy Scriptures, which should be chiefly read and thought over, since they teach us God's will and laws, are frequently neglected and even when read the sick are too apt to choose only their favourite chapters, or those they fancy will be most profitable to them. By thus doing they lose much of the meaning of the Bible. By taking a portion here and a piece there the sense is impaired, and frequently quite a wrong idea is gained, also many parts which are very instructive are never read at all. "All Scripture is given by inspiration of God, and is profitable for doctrine, for reproof, for correction, for instruction in righteousness, that the man of God may be perfect, thoroughly furnished unto all good works." Therefore we should read *all* Scripture lest we leave out what is profitable for our soul's health; in other words, we neglect just that wholesome purging medicine, that healing balm, that strengthening tonic which are needful for our cure.

It is very desirable that every one should have some plan of reading, for it is found to be a great help, especially to those who are shut off from other ordinary helps in public worship. Let us try and suggest some few rules which shall prevent us wasting our energies in turning over the Book till we have hit on something which has caught our eye as familiar, but far removed, probably, from the subject of our last reading. Reading stray chapters and verses will not answer the same end as going straight through from beginning to end. What notion would you get of a story book if you read a page and then skipped three or four and then looked to the end and finally glanced at the beginning? You might have a general notion of what it was about, but to be sure of anything in particular would be quite out of the question. To avoid this the Church has given a very good rule, and there can scarcely be a better one, for if we read the Psalms and Lessons daily, as far as our strength permits, we shall go through the Old Testament once and the New Testament three times every year and the Psalms twelve times. By this means we should get to know them thoroughly, and would soon find how wonderfully each Lesson and each Psalm was fitted to our particular wants, and would feel their teaching was given us by God and not chosen by ourselves. We shall also have the happiness of knowing we are not reading alone, but that hundreds of our fellow creatures, all members of the same Church, are using the same chapters.

You need not fear, this kind of reading may become a mere form—that would be a mistake, for it will grow less and less so, and the comfort will be so great that you will come to look on the hours so spent as the happiest part of your life.

